# Inguinal kimura disease misdiagnosed as Castleman disease: A case report and analysis of ultrasonographic features

**DOI:** 10.1016/j.radcr.2026.08.078

**Published:** 2026-09-18

**Authors:** Jiajue Tan, Lina Zhao, Yueyao Zhao, Yayun Zhou, Nan Chen, Bei Zhang

**Affiliations:** a Guizhou Medical University, Guiyang, China; b Department of Ultrasound Center, The Affiliated Hospital of Guizhou Medical University, Guiyang, China

**Keywords:** Kimura disease, Inguinal region, Ultrasonography, Eosinophilia, Castleman disease, Case report

## Abstract

Kimura disease (KD) is a rare chronic inflammatory proliferative disorder. Inguinal involvement is particularly uncommon, and its clinical and imaging appearances may mimic lymphoproliferative disease. We report a 38-year-old man (body mass index, 29.4 kg/m²) with a pruritic right inguinal mass present for more than 5 years. A previous diagnosis of Castleman disease (CD) had led to chemotherapy and surgery without satisfactory control. Ultrasonography showed a 70 × 32 × 46 mm heterogeneous hypoechoic subcutaneous mass containing scattered internal cord-like hyperechoic bands. Color Doppler imaging demonstrated abundant central arborizing intralesional vascularity. The absolute peripheral blood eosinophil count was 3.77 × 10^9^/L (32.6%). Noncontrast computed tomography demonstrated the dominant right inguinal mass and additional nodal lesions. Histopathologic examination after complete excision showed lymphoid follicular hyperplasia, extensive interfollicular eosinophilic infiltration, and eosinophilic microabscesses, confirming KD. This case expands the sonographic spectrum of inguinal KD and suggests that the combination of marked eosinophilia, internal cord-like hyperechoic bands, and central arborizing vascularity may help distinguish KD from CD before surgery.

## Introduction

Kimura disease (KD) is a rare chronic inflammatory disorder of uncertain etiology, generally associated with an abnormal T-helper 2 immune response [1,2]. It predominantly affects young Asian men and usually presents as painless subcutaneous masses or salivary-gland enlargement in the head and neck. Peripheral eosinophilia and elevated serum immunoglobulin E (IgE) are common, while histopathology shows lymphoid follicular hyperplasia, vascular proliferation, and extensive eosinophilic infiltration [[3], [4], [5]].

At atypical sites such as the inguinal region, KD lacks specific clinical and imaging findings and may be confused with lymphoma, metastatic lymphadenopathy, soft-tissue tumors, or Castleman disease (CD) [6,[7], [18]]. To our knowledge, few reports have specifically described ultrasonographic characteristics of inguinal KD that may assist differentiation from CD. We therefore correlate the clinical, laboratory, ultrasound, computed tomography (CT), and pathologic findings in a long-standing inguinal KD case initially diagnosed as CD, with particular emphasis on radiologic clues.

## Case report

### Medical history and physical examination

A 38-year-old man was admitted on January 9, 2026, for a pruritic right inguinal mass present for more than 5 years. Five years earlier, he had noticed hard, poorly mobile masses in the right inguinal region and right upper extremity, accompanied by generalized lymphadenopathy, fatigue, and intermittent low-grade fever. Histopathologic examination at another hospital was reported as hyaline vascular-type CD. Chemotherapy was administered (regimen unavailable), but the masses did not decrease appreciably. Resection of the right upper-extremity and inguinal masses was subsequently performed elsewhere; an egg-sized residual inguinal mass persisted and later became pruritic.

His body mass index was 29.4 kg/m². Apart from the prior mass-related treatment, relevant past medical history, family history, and regular medication history were negative. He had smoked approximately 10 cigarettes daily for more than 10 years, reported alcohol and seafood allergy, and denied tuberculosis or parasitic disease. Examination showed a healed right inguinal surgical scar and a firm, relatively well-demarcated, poorly mobile, nontender, nonreducible 6 × 5 cm mass. Several enlarged left inguinal and right axillary lymph nodes were also palpable.

### Laboratory examinations

The white blood cell count was 11.58 × 10^9^/L, with an absolute eosinophil count of 3.77 × 10^9^/L and an eosinophil percentage of 32.6%. Liver and renal function, coagulation studies, urinalysis, and electrocardiography showed no relevant abnormalities. Serum IgE, total IgG, IgG subclasses (including IgG4), and other immune-related tests were not performed.

### Imaging examinations

**Fig. 1:**
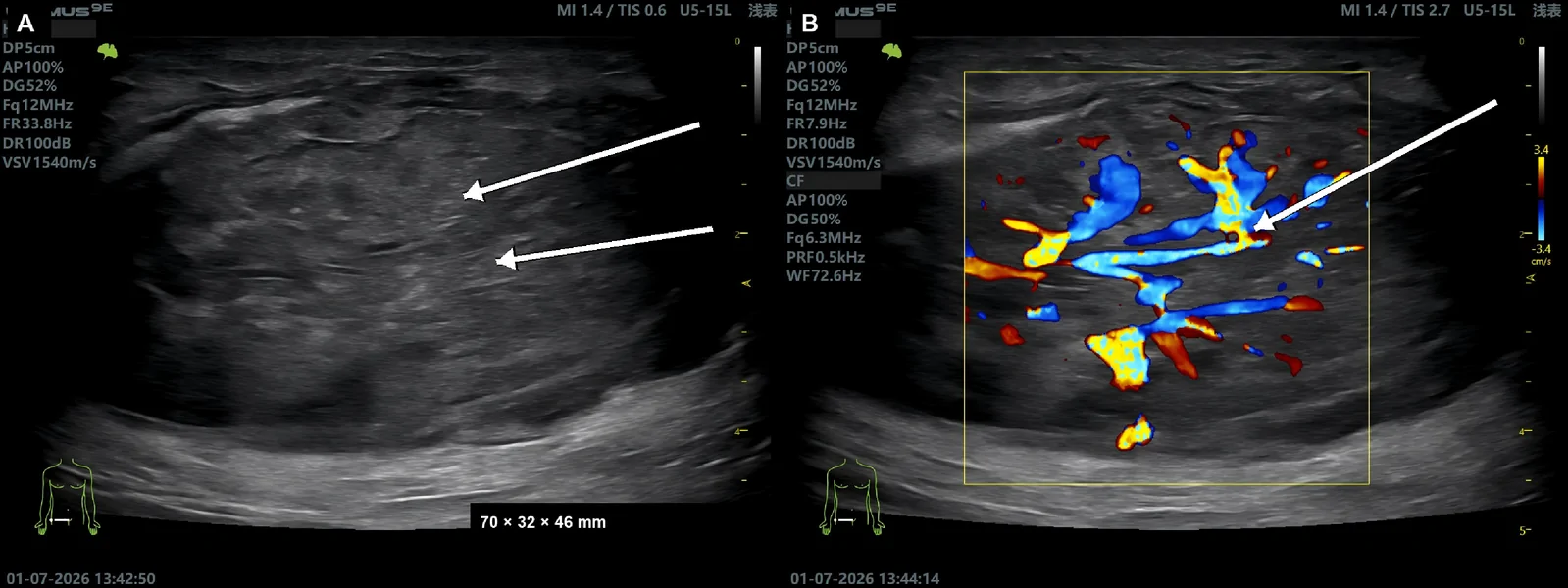
Ultrasonographic findings of the right inguinal region. (A) Gray-scale ultrasonography showed a 70 × 32 × 46 mm heterogeneous hypoechoic mass; white arrows indicate representative internal cord-like hyperechoic bands. (B) Color Doppler imaging showed abundant intralesional flow; the white arrow indicates the central arborizing vascular pattern.

Ultrasonography was performed using an ULTIMUS 9E system with a U5-15L linear-array transducer operated at 12 MHz. Transverse and longitudinal gray-scale and color Doppler acquisitions demonstrated a 70 × 32 × 46 mm hypoechoic mass in the subcutaneous right inguinal region. The lesion was oriented parallel to the skin, with a relatively clear boundary and regular contour. Its echotexture was coarse and heterogeneous, with scattered internal cord-like hyperechoic bands. No definite posterior acoustic enhancement or shadowing was identified. Color Doppler imaging showed abundant central arborizing intralesional flow. The surrounding subcutaneous tissue was thickened and demonstrated increased echogenicity (Fig. 1). Compressibility was not documented; elastography and spectral Doppler were not performed.

**Fig. 2:**
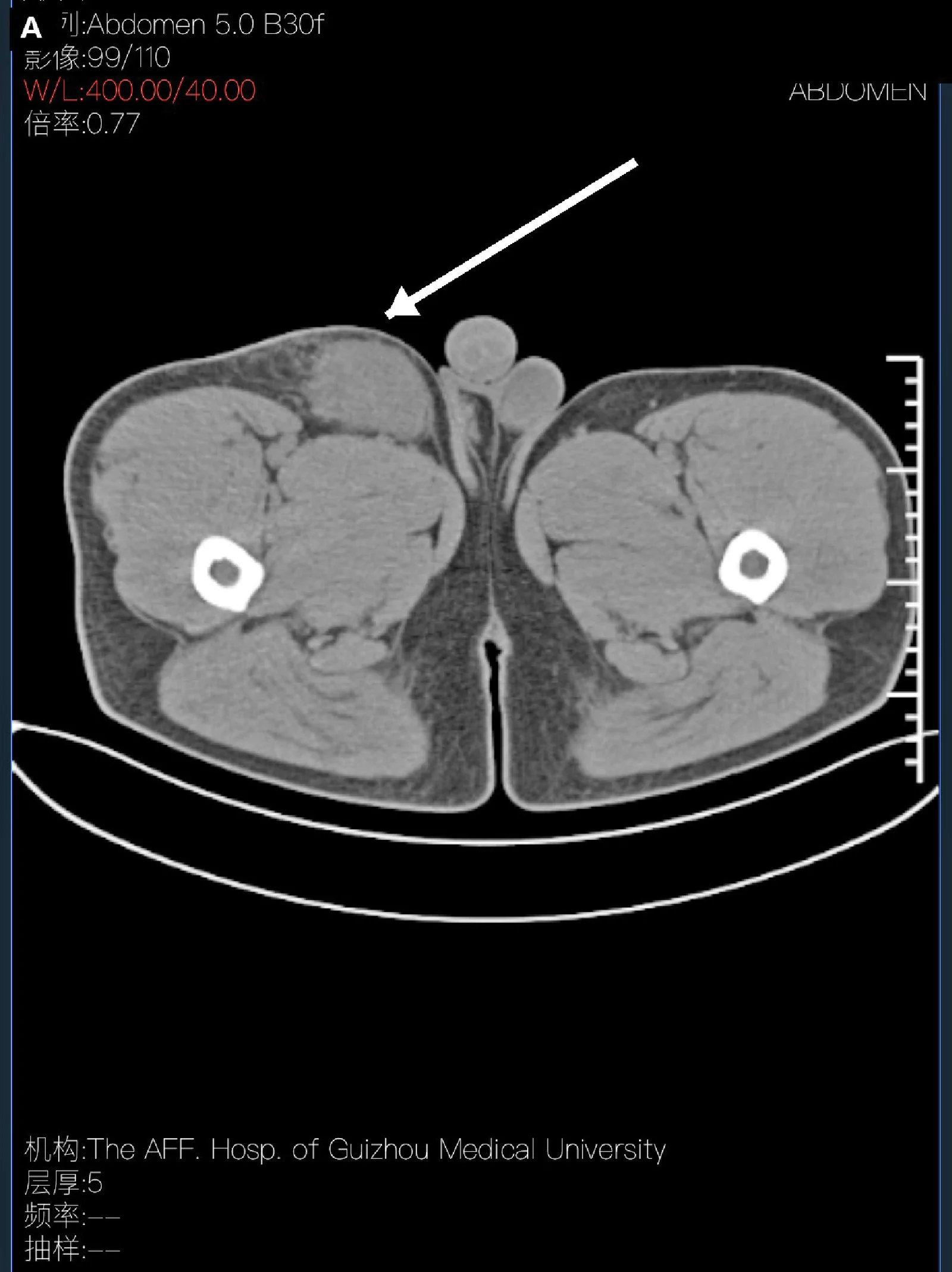
Axial noncontrast CT image of the inguinal region. The white arrow indicates the dominant right inguinal subcutaneous soft-tissue mass. The sagittal image from the original submission was removed because the lesion was incompletely included and poorly centered.

Noncontrast CT demonstrated multiple soft-tissue-attenuation lesions in the anteromedial right thigh/right inguinal region, along both iliac vascular chains, in the para-aortic region, and in the left inguinal region. The dominant right inguinal lesion measured approximately 58 × 39 mm and was centered in the subcutaneous tissue anteromedial to the proximal thigh. Adjacent fat planes were partially blurred with mild stranding, but there was no definite osseous destruction, deep muscular invasion, or vascular encasement on the available images. Enhancement could not be evaluated because intravenous contrast was not administered. In the context of the prior diagnosis, CD remained the leading CT interpretation (Fig. 2).

### Surgery and pathological findings

The right inguinal mass was excised under general anesthesia. It involved the subcutaneous and superficial fascial layers, measured approximately 8 × 5 × 4 cm, was firm and encapsulated, and had a relatively clear plane from adjacent tissue. Its medial aspect was closely related to the great saphenous vein. The lesion and adjacent small nodules were completely removed for pathologic examination.

**Fig. 3:**
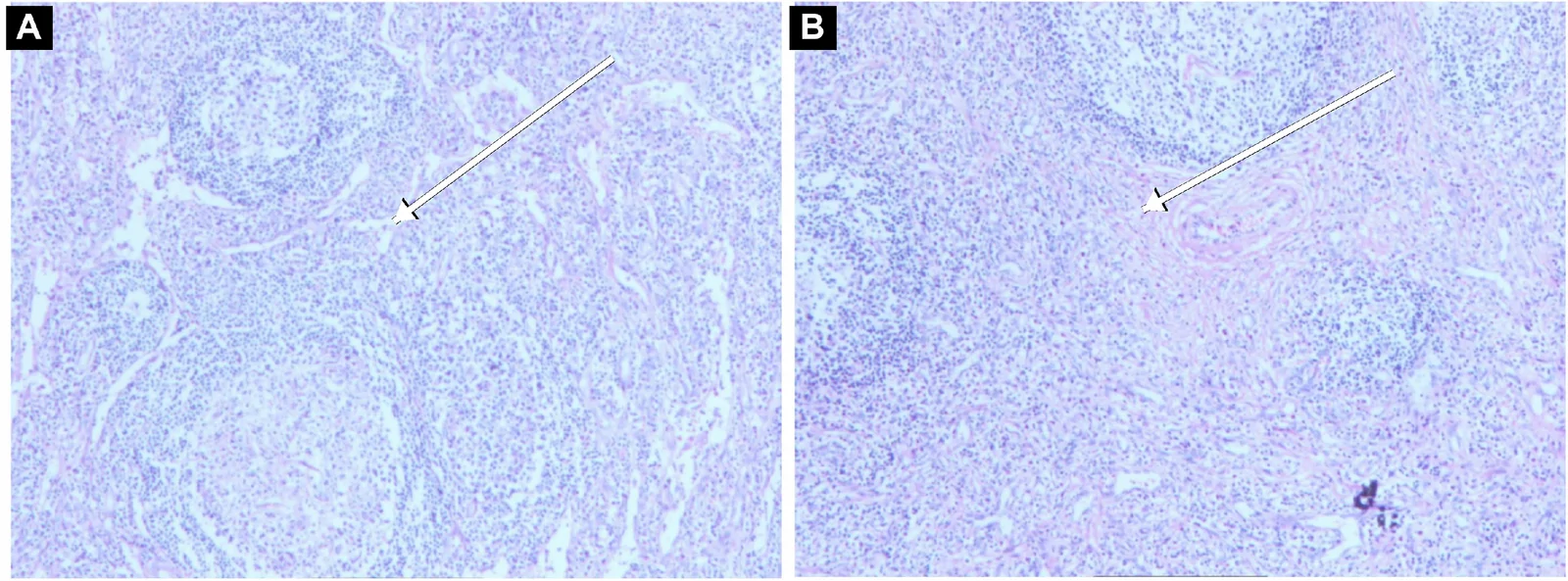
Postoperative histopathologic findings (hematoxylin and eosin stain, ×100). (A) White arrow indicates representative lymphoid follicular hyperplasia. (B) White arrow indicates extensive interfollicular eosinophilic infiltration with eosinophilic microabscess formation.

Hematoxylin and eosin-stained sections (×100) showed largely preserved lymph-node architecture, marked lymphoid follicular hyperplasia, interfollicular expansion with extensive eosinophilic infiltration, and eosinophilic microabscess formation, establishing the diagnosis of KD (Fig. 3). Immunohistochemistry, including IgG4, CD21, CD23, CD3, CD20, CD138, and Ki-67, was not performed.

### Postoperative recovery and follow-up

On postoperative day 6, the absolute eosinophil count decreased to 1.44 × 10^9^/L and the eosinophil percentage to 11.8%. The patient recovered well. At 6-month follow-up, pruritus had resolved and no local recurrence was identified.

## Discussion

The principal diagnostic challenge was the atypical inguinal location combined with multifocal lymphadenopathy, hypervascularity, chronicity, and an anchoring prior diagnosis of CD. KD and CD can both present as slowly enlarging painless masses and can share lymphoid follicular and vascular proliferation. However, KD more often occurs in young or middle-aged Asian men with head-and-neck or subcutaneous lesions, pruritus, peripheral eosinophilia, and elevated IgE. CD is a rare lymphoproliferative disorder that may arise wherever lymphoid tissue is present; unicentric disease is often asymptomatic, whereas multicentric disease may cause constitutional symptoms and multiorgan involvement. In our patient, marked eosinophilia and the poor response to prior CD-directed treatment were strong reasons to reconsider the diagnosis. IgE was not measured, so the diagnostic interpretation did not rely on an assumed elevation. A recent 53-patient series reported male predominance (88.7%), pruritus in 52.8%, head-and-neck involvement in 83.0%, and a mean eosinophil count of 2.09 × 10^9^/L, reinforcing the value of demographic and laboratory context [5]. Persistent marked hypereosinophilia also warrants clinical attention: a 2026 report described KD coexisting with cerebral autosomal dominant arteriopathy with subcortical infarcts and leukoencephalopathy (CADASIL) and recurrent cerebral infarction, although a causal relationship between KD and the neurologic events was not established [8].

Imaging findings should be interpreted as patterns rather than isolated signs (Table 1). Subcutaneous or salivary-gland KD often appears irregular, ill-defined, and heterogeneously hypoechoic, with coarse reticular, wool-ball-like, wood-grain-like, or cord-like echogenic structures [21]; nodal KD may instead remain oval, well defined, and show hilar or mixed vascularity. CD more commonly forms an oval or round, sharply marginated, sometimes encapsulated hypoechoic mass with internal linear or punctate echogenic septa, calcification, abundant flow, and a large penetrating vessel [[9], [10], [11], [12]]. A 2025 KD case report and scoping review found that imaging descriptions were heterogeneous and relatively sparse: CT most often identified parotid or neck masses, MRI identified parotid or facial masses, and ultrasound generally showed hypoechoic lesions [6]. Thus, internal echogenic bands are not pathognomonic for KD; their value in this case arose from their combination with central arborizing flow, surrounding inflammatory change, marked eosinophilia, and discordance with the previous treatment response.

**Table 1:** Imaging-focused comparison of Kimura disease and Castleman disease.

Feature	Kimura disease (KD)	Castleman disease (CD)
Typical distribution	Usually head and neck, salivary-gland region, or subcutaneous soft tissue; inguinal disease is rare. Nodal and extranodal patterns occur [6,5,13].	Any lymphoid-bearing region; mediastinal, retroperitoneal, mesenteric, cervical, axillary, pelvic, and occasionally inguinal sites.
Number/clinical pattern	May be solitary or multifocal; chronic painless mass, often with pruritus or skin change [5].	Unicentric disease is usually a solitary slow-growing mass without systemic symptoms; multicentric disease causes generalized lymphadenopathy and may produce constitutional or multiorgan manifestations.
Shape and boundary on ultrasound	Salivary-gland/subcutaneous type: often irregular and ill defined. Nodal type: may be oval, regular, and well defined.	More often oval or round, regular, sharply defined, and encapsulated; lobulation can occur [9,11,12].
Gray-scale echogenicity	Heterogeneous hypoechoic tissue, often coarse; hypoechoic lesions predominate in published ultrasound descriptions [6].	Markedly hypoechoic or hypoechoic; homogeneous or mildly heterogeneous, although heterogeneity is frequent [9,12].
Internal echogenic structures	Coarse reticular, wool-ball-like, wood-grain-like, or cord-like hyperechoic bands, reflecting fibrosis/inflammatory architectural disorganization.	Fine linear or punctate echogenic septa; calcification may occur [[9], [10], [11], [12]].
Doppler vascularity	Often abundant; subcutaneous lesions may show central arborizing flow. Nodal lesions may preserve hilar or mixed flow.	Usually abundant with peripheral, hilar, or mixed distribution; a prominent penetrating feeding vessel may be present [[9], [10], [11], [12]].
CEUS	Limited evidence; may show hyperenhancement, but no validated disease-specific pattern.	Frequently high or isoenhancement, sometimes progressing from the periphery toward the center [11].
CT/MRI	May be infiltrative or ill defined. Orbital KD has shown iso- to slightly low T1 signal, heterogeneous slightly high T2 signal, T2-low fibrous septa, and marked heterogeneous enhancement [13].	Usually well-defined on CT/MRI, with mild hypo-/isodensity on nonenhanced CT, low-to-isointense T1 signal, iso-to-hyperintense T2 signal, marked enhancement, and possible hypertrophied vessels or calcification [14].
PET/CT	FDG uptake may occur and is nonspecific [6].	Multicentric disease may show relatively symmetric, nonconfluent, moderately hypermetabolic lymphadenopathy with systemic involvement [15].
Laboratory clues	Peripheral eosinophilia and elevated serum IgE are common; renal involvement/proteinuria can occur [5,8].	Marked eosinophilia is uncommon; inflammatory abnormalities are more typical of multicentric disease.
Pathology	Follicular hyperplasia, expanded interfollicular zones, extensive eosinophilic infiltration, eosinophilic microabscesses, and folliculolysis [6,5,13].	Hyaline vascular, plasma-cell, and mixed variants. Hyaline vascular lesions show vascularized/regressed germinal centers and hyalinized vessels; plasma-cell lesions show interfollicular plasma-cell sheets.

Imaging appearances overlap and vary with lesion site, disease extent, and histopathologic subtype; tissue diagnosis remains definitive.

CEUS, contrast-enhanced ultrasonography; FDG, fluorodeoxyglucose; PET/CT, positron emission tomography/computed tomography.

The Doppler difference has a plausible histologic basis. In KD, diffuse interfollicular inflammation and small-vessel proliferation may produce branching central channels, while fibrosis and disorganized inflammatory architecture may generate coarse echogenic bands. In hyaline vascular CD, capillary proliferation within and between follicles, hyalinized vessels, and fibrous septa may yield a conspicuous feeding vessel with peripheral or mixed perfusion and finer linear echoes [9,12]. Contrast-enhanced ultrasonography (CEUS) may show high or isoenhancement in CD, sometimes progressing from the periphery toward the center [11]; however, overlap with hypervascular KD limits specificity. CEUS was not performed in this patient.

Cross-sectional imaging can define extent and guide surgery but is usually not diagnostic. KD on CT or magnetic resonance imaging (MRI) may be infiltrative or ill defined, with variable T2 signal from fibrosis and prominent enhancement or flow voids from vascularity. In a 2026 orbital KD series, lesions were iso- to slightly hypointense on T1-weighted images and heterogeneously slightly hyperintense on T2-weighted images; strip-like T2-hypointense septa and marked heterogeneous enhancement were attributed to coexisting fibrosis and active inflammation [13]. Although these observations are site-specific, they support MRI for mapping lesion extent rather than establishing a definitive diagnosis. CD commonly appears as a well-defined, mildly hypodense or isodense mass on nonenhanced CT, with intermediate-to-marked enhancement after contrast administration; MRI typically shows low-to-isointense T1 signal, iso-to-hyperintense T2 signal, diffusion hyperintensity, and marked enhancement. Hypertrophied vessels and calcification are useful additional clues [14]. Both diseases may demonstrate fluorodeoxyglucose uptake on PET/CT, so uptake intensity alone is nonspecific; in multicentric CD, PET/CT can demonstrate relatively symmetric, nonconfluent, moderately hypermetabolic nodal disease and systemic involvement [6,15]. The absence of contrast-enhanced CT, MRI, CEUS, and PET/CT in our patient limited preoperative characterization. Lymphoma should be considered when multiple markedly hypoechoic nodes lose their fatty hila [19]; metastatic nodes more often show cortical asymmetry, necrosis, calcification, or a known primary tumor; and soft-tissue sarcoma is favored by a heterogeneous invasive mass with necrosis, fascial transgression, or rapid growth. None of these features supersedes tissue diagnosis when imaging and laboratory findings conflict.

Definitive diagnosis rests on histopathology. KD is characterized by follicular hyperplasia, expanded interfollicular zones, extensive eosinophilic infiltration, eosinophilic microabscesses, and sometimes folliculolysis. Hyaline vascular CD classically shows regressed or vascularized germinal centers, concentric mantle-zone lymphocytes, and prominent hyalinized vessels; plasma-cell CD features sheets of interfollicular plasma cells. Complete excision established the diagnosis and relieved symptoms in this patient [20]. Because KD may recur, follow-up with local ultrasonography and peripheral eosinophil counts is appropriate; immune markers may be added if clinically indicated [16]. A 53-patient series reported an overall recurrence rate of 60.4% and favorable responses to mepolizumab in 3 refractory cases [5], whereas a 2026 orbital series observed recurrence in 2 of 7 patients with follow-up and emphasized structured clinical and imaging surveillance [[13], [17]]. These findings support long-term monitoring; biologic therapy should be reserved for appropriately selected recurrent or refractory disease.

### Patient perspective

The patient reported resolution of pruritus and no palpable mass at the original right inguinal site after surgery. At the latest follow-up, he had no additional discomfort.

## Conclusion

Recognition of internal cord-like hyperechoic bands and central arborizing vascularity in conjunction with marked eosinophilia may improve the preoperative diagnosis of inguinal KD and reduce misdiagnosis as CD. Imaging findings remain overlapping, and histopathologic confirmation is essential.

## Ethics statement

Ethics approval was waived by the Institutional Review Board of the Affiliated Hospital of Guizhou Medical University because this report describes a single anonymized clinical case.

## Data availability

Data sharing is not applicable because no datasets were generated or analyzed beyond the clinical case data reported in this article.

## Declaration of generative AI and AI-assisted technologies in the writing process

During the preparation of this work, the authors used OpenAI’s ChatGPT and Codex to assist with English-language polishing, manuscript organization. After using these tools, the authors reviewed and edited the content as needed and take full responsibility for the content of the publication.

## Author contributions

Conceptualization: Jiajue Tan, Lina Zhao, Yueyao Zhao, Yayun Zhou, Nan Chen, Bei Zhang. Data curation: Jiajue Tan. Investigation: Jiajue Tan. Methodology: Lina Zhao, Yueyao Zhao, Yayun Zhou. Writing—original draft: Jiajue Tan, Lina Zhao. Writing—review and editing: Lina Zhao, Yueyao Zhao, Yayun Zhou, Nan Chen, Bei Zhang. All authors read and approved the final manuscript.

## Patient consent

Informed consent was obtained from the patient for publication of this case report and any accompanying images.
